# “White Cord Syndrome” of Acute Tetraplegia after Anterior Cervical Decompression and Fusion for Chronic Spinal Cord Compression: A Case Report

**DOI:** 10.1155/2013/697918

**Published:** 2013-03-04

**Authors:** Kingsley R. Chin, Jason Seale, Vanessa Cumming

**Affiliations:** ^1^Charles E. Schmidt College of Medicine, Florida Atlantic University and Institute for Modern & Innovative Surgery (iMIS), 1100 W. Oakland Park Boulevard, Suite No. 3, Fort Lauderdale, FL 33311, USA; ^2^iMIS Surgery, 1100 W. Oakland Park Boulevard, Suite No. 3, Fort Lauderdale, FL 33311, USA; ^3^LES Society, 300 E. Oakland Park Boulevard, Suite 502, Fort Lauderdale, FL 33334, USA

## Abstract

Paralysis is the most feared postoperative complication of ACDF and occurs most often due to an epidural hematoma. In the absence of a clear etiology, inadequate decompression or vascular insult such as ischemia/reperfusion injury are the usual suspects. Herewith we report a case of complete loss of somatosensory evoked potentials (SSEPs) during elective ACDF at C4-5 and C5-6 followed by postoperative C6 incomplete tetraplegia without any discernible technical cause. A postoperative MRI demonstrated a large area of high signal changes on T2-weighted MRI intrinsic to the cord “white cord syndrome” but no residual compression. This was considered consistent with spinal cord gliosis with possible acute edema. The acute decompression of the herniated disc resulted in cord expansion and rush-in reperfusion. We postulate that this may have led to disruption in the blood brain barrier (BBB) and triggered a cascade of reperfusion injuries resulting in acute neurologic dysfunction. At 16 months postoperatively our patient is recovering slowly and is now a Nurick Grade 4.

## 1. Introduction

Anterior cervical decompression and fusion (ACDF) are commonly performed procedures for conditions resulting in symptomatic nerve root and/or spinal compression anteriorly. ACDF is associated with favorable fusion rates and good outcomes [1–4]. Paralysis is extremely rare but the most feared among postoperative complications [5]. Paralysis is most often due to an epidural hematoma, but in the absence of clear etiology, inadequate decompression or vascular insult such as ischemia/reperfusion injury possibly due to oxygen-derived free radical damage [6–8] are the usual suspects. Oxygen-derived free radicals seem implicated in neuronal injury as are mitochondria-dependent apoptosis, TNF-*α* production, and specific phospholipid signaling cascades [9–11].

We report a case of complete loss of somatosensory evoked potentials (SSEPs) during elective ACDF at C4-5 and C5-6 followed by postoperative C6 incomplete tetraplegia without any discernable technical cause. We describe this occurrence as a “white cord syndrome” because of the postoperative appearance of a large area of cord edema behind the massive herniated disc seen on sagittal T2-weighted magnetic resonance imaging (MRI). The MRI appearance of the preoperative and postoperative management, outcome, and proposed pathophysiology of this syndrome are discussed.

## 2. Case Presentation

**Figure 1:**
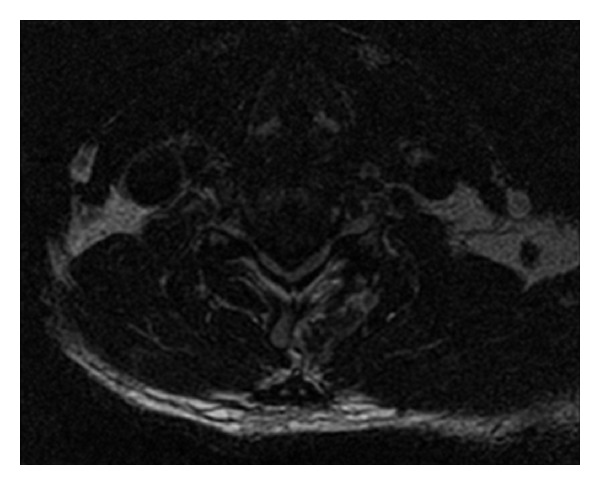
Preoperative axial T2-weighted MRI showing severe C5-6 cord compression by a massive disc herniation.

**Figure 2:**
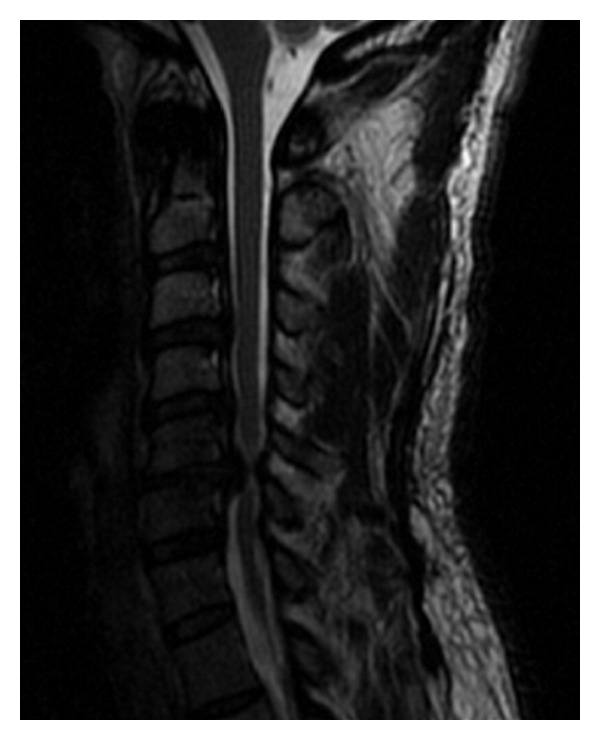
Preoperative MRI sagittal showing large area of high signal intensity centered behind the massive C5-6 herniated disc.

A 59-year-old male patient was referred to us with a MRI diagnosis of a large C5-6 herniated disc causing severe cord compression, neck pain, radiculomyelopathy, and ataxia. The patient gave a seven-month history of neck pain with shoulder radiation, pain in the lower back radiating to both legs, and balance difficulties. At initial examination cervical range of motion was limited (flexion, extension, left and right rotation), and he reported pain specifically at the end of extension. A markedly positive Hoffman's sign (right > left) was elicited. He was assessed as Nurick Grade 3 at this initial visit and diagnosed with cervical radiculomyelopathy. MRI demonstrated a massive herniated nucleus pulposus at C5-6 with severe cord compression and myelomalacia at the level of the herniated disc (Figures 1 and 2).

The patient underwent C4-5 and C5-6 ACDF. Intraoperative electroneurophysiological monitoring was performed continuously throughout the surgical procedure. This involved spinal cord monitoring with somatosensory recordings (somatosensory evoked potentials (SSEPs)), nerve activity monitoring, cortical recordings, and motor evoked potentials (MEPs) are performed.

**Figure 3:**
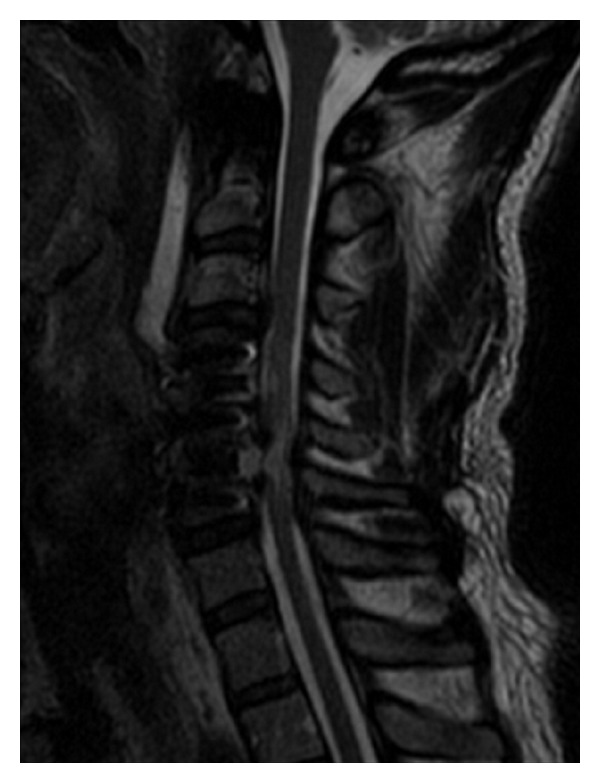
Immediate postoperative MRI after primary C4-5, C5-6 ACDF demonstrates residual C5 compression.

An interbody PEEK cage (8 mm) (Eminent Spine, Texas) was placed at the C5-6 level. After cage placement at C5-6 the surgical team was informed about diminished MEP signals. The cage was removed without any changes and so replacing and monitoring continued. The surgical procedure was repeated at the C4-5 level with partial corpectomies, discectomy, and placement of an 8 mm interbody PEEK cage. It was then reported that the diminished signal recordings were attributed to marked dysfunction of spinal cord conduction pathways. At this time there were no measureable MEPs, and so the PEEK cages were removed. The signal did not return with removal of the cages, so the patient was awoken and asked to move his limbs; he was able to move his arms only at the time. The procedure was urgently completed by replacing the interbodies and placing an anterior plate (SpineFrontier Inc., InVue plate, Beverly, MA, USA) between C4, C5, and C6. Postoperatively the patient demonstrated a C6 incomplete tetraplegia. MRI raised concerns about residual bony compression at C5 (Figure 3). MRI and CT were done emergently postoperatively which raised concern for residual bony compression mostly behind the C5 body and the edges of C4 and C6. The surgeon was concerned about a vascular/reperfusion phenomenon given the acute decompression of a severely and chronically compressed spinal cord. The patient was urgently returned to the operating room for more extensive decompression, and corpectomy at C5, to provide the cord with as much room possible for recovery. During the revised procedure SSEP responses from the left lower limb were noted to be poorly reproduced and to have very low amplitude but were noted to be present in the right lower limb. Transcranial MEPs were present and reproducible from upper limb but not lower limb muscle groups. This was deemed encouraging.

There were no cerebrospinal fluid (CSF) leaks or increased blood loss reported intraoperatively during this patient's surgical procedures. In fact, from a surgical perspective the procedures were quite uneventful and uncomplicated apart from the complete loss of SSEPs. 

Hydrocortisone 100 mg was given intravenously intraoperatively during the revised procedure. Subsequently, the patient was placed on acute inpatient rehabilitation in the intensive care unit for his C6 incomplete tetraplegia and an acute spinal cord injury steroid protocol [12] was begun and tapering intravenous dexamethasone was added. 

**Figure 4:**
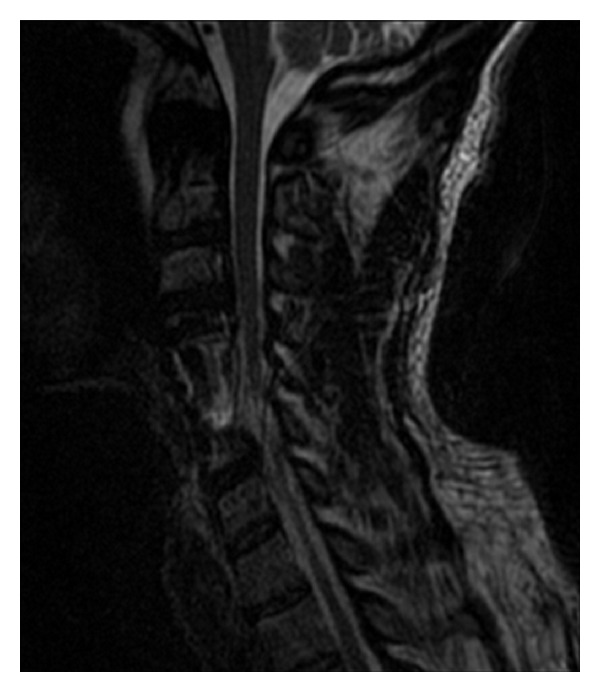
Day 3 postoperative MRI demonstrates more clearly the spinal cord edema intrinsic to the cord—a “white cord syndrome.”

At day 3 a postoperative MRI demonstrated a large area of high signal changes on T2-weighted MRI intrinsic to the cord (“white cord syndrome”) but no residual compression (Figure 4). Following intravenous administration of gadopentetate dimeglumine contrast at MRI postoperatively there was no pathologic enhancement of the spinal cord lesion, and therefore the hyperintense changes involving the cervical spine at the C5 level were considered consistent with spinal cord edema (Figure 3). When compared to the preoperative MRI, it was clearly present but partially masked by the large herniated disc. Day 2 postoperatively the patient was returned to the operating room to investigate any SSEP changes to and to see if there was continued recovery. There were no changes to SSEP compared with intraoperatively. The decision was then made to continue steroids and follow the patient clinically with serial MRIs. Within two days the patient moved both upper limbs and had 3/5 power in the toes.

**Figure 5:**
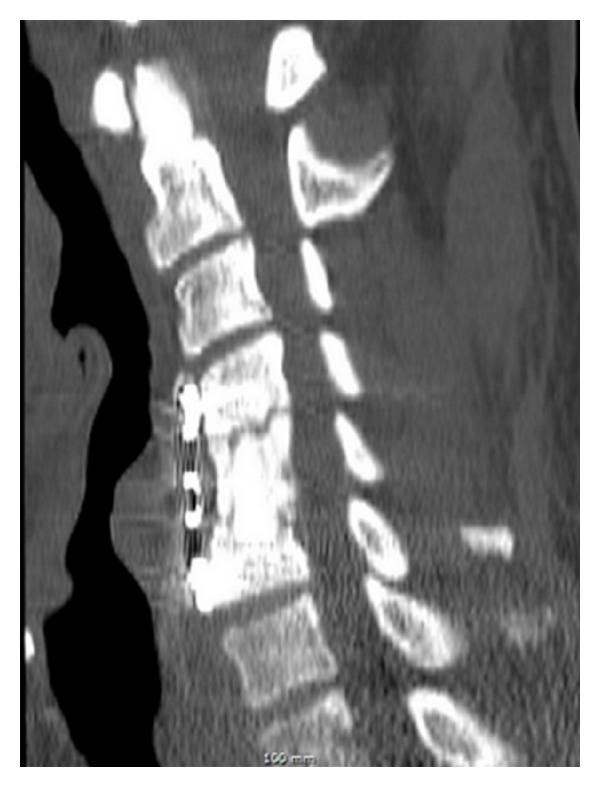
Sagittal CT reconstruction at the latest followup shows graft consolidation confirming fusion.

**Figure 6:**
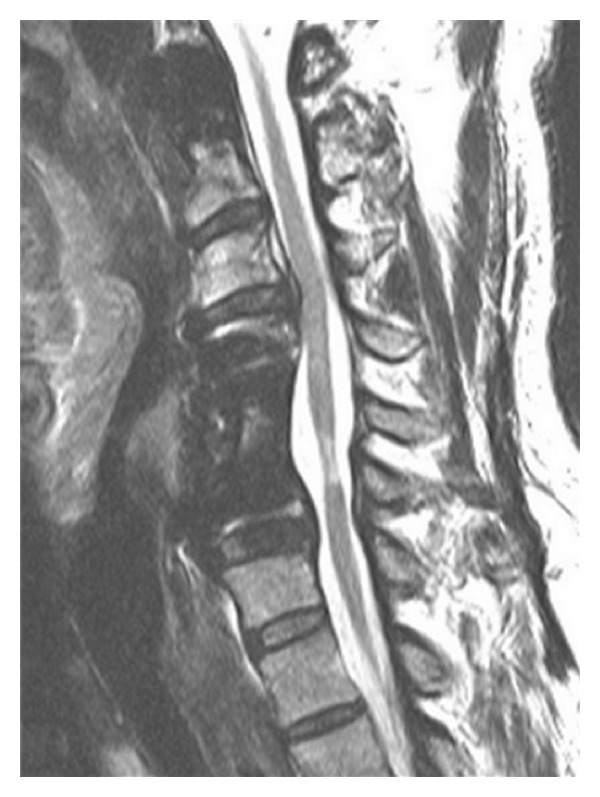
Sagittal T2-weighted MRI at the latest 16-month followup showing a persistent “white cord syndrome.”

The patient was discharged to an inpatient rehabilitation facility at day four postoperatively without any signs of recovery. His bulbocavernosus reflex was equivocal. Gradually over the next two months, his bilateral upper limb strength improved to full strength except for 3+/5 left finger flexion, extension, and interossei. His right lower extremity also improved to full strength. His left lower extremity strength lagged substantially behind with grade 3/5 hip flexor, adductor, and abductor strength. At the latest 16-month followup he was still weak in his left finger flexion (3/5), finger extension and interossei (4/5), and his left lower limb with 5−/5 hip abduction power, 4/5 quadriceps and hamstrings, and 2/5 in all other muscle groups. Reflexes were 2+ except 1+ right triceps and 3+ at the left knee with sustained left ankle clonus. Sensation was decreased on the left hand, leg, and thigh. At this visit a Nurick Grade 4 and ASIA score D were assessed. A slight improvement in standing and walking was noted, but he still required an assistive frame to get around the house and a wheelchair outside (Table 1). Radiography and MRI reviewed at this visit confirmed fusion but demonstrated persistent gliosis (Figures 5 and 6).

## 3. Discussion

In this patient, a massive herniated disc seemed to have compressed the cord chronically and produced a large area of cord edema, but the patient had compensated. Over time his radiculomyelopathic symptoms progressed and he sought surgical treatment. The acute decompression of the herniated disc resulted in immediate cord expansion within the open canal space, and the compressed segment of the cord exposed to a rush in blood supply. We postulate that this sudden cord expansion and reperfusion may have lead to disruption in the blood brain barrier (BBB), or in the blood spinal cord barrier, and triggered a cascade of reperfusion injury resulting in acute neurologic dysfunction at and below the C6 level. The MRI appearance on sagittal T2-weighted MRI and the clinical results of incomplete paralysis without a clear understanding of the pathophysiology of this condition led us to use the term “white cord syndrome.” 

A cohort study reported by Seichi et al. in 2004 followed more than one hundred patients with MRI three weeks postlaminoplasty to determine the frequency of swelling of the spinal cord with an intramedullary lesion and the possible mechanism of postoperative motor paresis of the upper extremity [13]. They reported a 6.1% incidence of postoperative abnormal expansion of the T2 high signal intensity area; of which 43% were asymptomatic. The upper motor paresis described in their cohort was strongly related to distal and diffuse type of postoperative paresis of the upper extremity without deterioration of lower motor function. This report was the only other description of similar MRI findings as what we experienced with our patient. That being said, our patient's presentation was more dramatic after ACDF and followed a different pattern of motor paresis from that described in the previously mentioned postlaminoplasty cohort.

Spinal cord ischemia/reperfusion injury appears contingent on oxygen-derived free radical damage [6–8], mitochondria-dependant apoptosis, TNF-*α* production, and specific phospholipid signaling cascades resulting in neuronal injury in human and animal models [9–11, 14–16]. It has been suggested that acute and chronic spinal cord ischemic injury may in fact induce the passage of blood borne or neurotrophic substances (specifically TNF- *α*) through the BBB past its saturation point [14, 16–18]. It appears that decoupling of astrocyte foot processes from endothelial cell surfaces inhibits tight junction function in the BBB [15, 19, 20]. Transport systems and ionic buffering would then be disrupted allowing worsened reperfusion injury upon decompression of a previously ischemic spinal cord.

To date substantial efforts have focused on the mitigation of spinal cord ischemic injury. These efforts have included surgical techniques (such as timing of surgical decompression, temporary shunts, or partial bypass), pharmacological interventions (such as methylprednisolone), and mechanical methods (e.g., hypothermia or drainage of cerebrospinal fluid) [18, 21–26]. More recently it has been suggested that potent antioxidants may also play a role in the management of spinal cord ischemic/reperfusion injury [7].

In our case described herewith, MRI performed at day 1 postoperatively demonstrated gliosis. However, there remains much debate about the clinical relevance of high signal intensity on the T2-weighted MR images. The debate extends to the reversibility of the spinal cord edema also [27]. It should be noted that the increased T2-weighted signal intensity was present even before the decompression, so demyelination may also be a possibility in this patient. We highlight these factors simply to reflect on the different possible reasons for the edema and the increased signal intensity.

We have presented this theory as to the pathophysiology behind this patient's intra- and postoperative complications as there was no clinically significant hematoma or CSF leakage reported that could have led to a pseudomeningocele and made a more plausible explanation of our findings.

In light of the proposed etiologies implicated in triggering this syndrome [6, 7, 9–11] the clinical presentation of this patient is instructive in raising awareness. The management of this “white cord syndrome” will conceivably include adequate surgical decompression and pharmacological treatment options [7, 18, 21–26]. However, patients and surgeons should be aware of the potential catastrophic results after a seemingly routine ACDF to treat a large herniated disc with severe and chronic cord compression. This patient had motor function return fairly rapidly within two months but slowed down substantially in the ensuing months. This may help the surgeon to advise patients. A full corpectomy is a good option in this scenario to ensure adequate decompression. Postoperative SSEP monitoring is an option to monitor recovery during the immediate postoperative period. The use of steroids should be individually weighed against the risks.

## Figures and Tables

**Table 1:** Rehabilitation assessment: American Spinal Injury Association (ASIA) scores.

	Immediate postoperative function	Hospital discharge	2-months postdischarge	16-month followup
ASIA motor score	B	C	D	D
